# Artificial intelligence in healthcare – Attitudes towards AI among Hungarian healthcare professionals

**DOI:** 10.1192/j.eurpsy.2025.1435

**Published:** 2025-08-26

**Authors:** Á. K. Magyary, N. Z. Mészáros, M. B. Szondy, Z. Demetrovics, Á. Zsila

**Affiliations:** 1 Doctoral School of Psychology; 2 Institute of Psychology, ELTE Eötvös Loránd University; 3 Institute of Psychology, Catholic University of Pázmány Péter, Budapest; 4 Institute of Psychology, Universsity of Pécs, Pécs; 5 MAZSIHISZ Charity Hospital, Budapest, Hungary; 6 Centre of Excellence in Responsible Gaming, University of Gibraltar, Gibraltar, Gibraltar; 7 College of Education, Psychology and Social Work, Flinders University, Adelaide, Australia

## Abstract

**Introduction:**

Artificial intelligence (AI) can potentially enhance healthcare professionals’ understanding of certain disorders, facilitating improved diagnosis, treatment, and prevention. Exploring potential psychological factors that can possibly influence healthcare professionals’ attitudes towards AI in their work is crucial to assist successful adoption and utilization of these technologies.

**Objectives:**

The possible role of burnout, perceived distress, and factors related to work circumstances on willingness to use AI were explored in this investigation.

**Methods:**

Attitudes towards artificial intelligence, perceived distress and factors related to work were assessed by using an online questionnaire. Participants (86 % women, M*age* = 46.9 years, SD = 11.3) were healthcare professionals recruited from Hungarian hospitals and healthcare institutions.

**Results:**

Linear regression analysis indicated that most participants (58%) were open to using AI in their work. Significant predictors of use were job satisfaction, work performance, and administrative workload. Higher burnout levels and perceived distress were not associated with attitudes towards AI.

**Conclusions:**

The present findings suggested that work-related environmental factors may have a greater predictive power in explaining the propensity to use AI in healthcare than individual psychological factors. However, the explanatory power of these factors in AI use was modest (7.5%), suggesting that future research should investigate further possible predictors of attitudes towards AI such as social factors.

**Disclosure of Interest:**

None Declared

